# Cellars in Dwellings

**Published:** 1879-01

**Authors:** 


### CELLARS IN DWELLING-HOUSES.

With a fact like the above staring one in the face, and in connection with another, that farmers generally make their cellars the winter and summer receptacles of every variety of vegetables and fruits, more or less of which are put away in a bruised, rotted, or unripe condition, and thus speedily become putrid, by acetous fermentation without the aid of much heat, it is apparent that these gases are constantly ascending, and must unavoidably impregnate every room in the house with a vitiated and unwholesome atmosphere; and in consequence of another known fact, and unfortunately almost universal, that the cellar being convenient and "out of sight" of visitors, is made the receptacle of all that is old and unseemly as well as of kitchen offal, by the laziness of bad housekeepers or unprincipled servants. For these considerations, it is clear that no cellar should be built under that part of a house which is to be occupied as a place to eat and sleep and live in, whether in town or country. The great cost of land in towns and cities may be some apology for having cellars underneath; but there is none for having cellars under a farm-house. Where there are already cellars, a great deal may be done toward preventing them from becoming the fruitful source of sickness and suffering; and if there is any obscure or slow disease in the family of any reader of this article, and a cellar is attached to the building, it is worth the experiment to secure the following "alterations" as to the cellar: let the cellar be emptied of every movable thing; let the walls and floor be thoroughly swept, and, if practicable, washed; and after being allowed to "air" for a week or two, have the ceiling plastered, the space between that and the floor having been filled with dry sand or gravel, or ashes or pulverized charcoal, not only to keep dampness and cold and any hurtful chance emanations coming up from below, but also, in case water should leak through the floors, it might easily pass through the sand, and thus perfect dryness be secured. The walls should be smoothly plastered, and the floor covered with a hard cement, thick, smooth, and strong; and both walls and ceiling should be well whitewashed twice a year; once a year, at least, the old whitewash should be scraped or swept off, before the new is applied. The best, because the cheapest and most universally available whitewash, is made as follows: put unslaked lime, that which is in the form of the original rock, in a vessel; pour boiling water on it until it is covered; place a cloth over the vessel so as to confine the most minute particles of the lime, they being the ones which most perfectly "penetrate" the surfaces to which the wash is applied, and consequently remain the longest. Subsequently dilute the wash to the consistence of thick cream, and apply it thoroughly and thickly, thus accomplishing two objects, a white, light-giving surface, having a "body," as painters term it, which is capable of absorbing, and thus rendering harmless the "bad" airs or gases which may be formed in the cellar.

Every partition and every shelf in a cellar should be made of smoothly planed boards, well covered with good white paint, thus preventing the accumulation of dust, and aiding in making the cellar light, cheerful, and clean, for the more light you can have, the better. Every cellar should be so contrived, that either by its grating or windows or doors, it may be easily and thoroughly ventilated, an hour or two at least every day in the year

It is scarcely necessary to remark, that if a cellar is liable at any time of the year, even for a few days, to have water rise and stand on the floor or even to have the floor a little wet, draining tiles should be put under it before the floor is "cemented." All shelves in a cellar should be so arranged that you can go all around them; it is not advisable to put any shelving against a cellar-wall; and if all the shelves are suspended from the ceiling, so much the better on several accounts, not the least of which is that more "floor-room" is thus obtained.

When a house is to be erected in a new locality, and it has been wisely determined to have the cellar off from the family building, but yet to be easily accessible from the kitchen without having to go "out of doors"—say under the kitchen itself or under the wood-house, or simply under the ground, its roof being a part of the front yard or garden, if you please, but so covered over with soil and grass, bushes, etc., that it would not be known to be there — the next point is to arrange that the foundation of the house should be on a rock, at least three feet deep, and on a spot descending, if possible, in every direction. The walls of the house should be at least two feet above the surface of the earth, crevices having been left at intervals on each side, so as to admit a free circulation of air, but not large enough to admit mice. There should be an open ditch all around the inside of the wall, as a drain to any dampness, with a sufficient descent, at least at one point, to insure the drain to be passed off.

It is well to plaster a foundation wall inside and out, and to have every stone well laid in a good mortar, not being sparing of lime or sand in its preparation. Too much "loam," or common dirt, is generally used, so that the mortar crumbles to powder, has no tenacity, no binding power, instead of hardening and becoming a part of the wall itself.

The space between the lower edge of the joists of the ground floor and the upper edge should be filled with dry sand, ashes, or, which is much better, charcoal, for the three-fold object of, first, keeping the lower floor dry; second, keeping it warmer in winter; third, absorbing any deleterious gases which might arise from the ground. As to the materials for building, each locality has its peculiar conveniences, but it should not be forgotten that wooden buildings are best for the country, because they are dryer, and consequently more healthful.

The best kind of roof for a country house is the old-fashioned steep roofs, with a "comb" in the center; with no "hips" or dormer windows; these may make a building more picturesque, but they so generally leak, that a plain, steep, shingled roof is safer, more economical, and more universally available.

As to the shape and size and hight of the rooms, each builder must decide for himself, according to his taste and the length of his purse. A square building gives most room for the same money; and a broad hall in the center of the building affords greater advantages than any other arrangement.

"High ceilings," as they are called, are now much the fashion; but they are more costly in the first place, and occasion an unnecessary waste of fuel ever thereafter; they are commended for their spaciousness; but they sometimes give a barn-like appearance to a house, and are never so cosy as rooms which are not quite so high.

Winding stairs are objectionable everywhere, but especially in the country, where persons rise by daylight or sooner, and where there are old persons or young children; as in haste or darkness there is danger of falling, and breaking or disjointing the limbs or neck.

It is a great saving in the cost of furniture, if, in the erection of new buildings, and in the modification of old ones, large, light, and roomy closets are plentifully supplied, and with them shelves, hooks, and drawers. Many persons in the country when "dressed," show bad housekeeping and characteristic slovenliness, by having their outer garments marked with innumerable "creases," showing that they have been thrown negligently into a drawer, and allowed thus to remain from one "going-out" to another. The outer dresses of both sexes should be hung up in closets, protected by doors from dust; and to this end, every farm-house should have a great abundance of closet-room. These closets should be always large, and all the doors should be hinged within two or three inches of the wall, so that there may be no dark corners for the collection of dust or other improper thing, or for the hiding of what is valuable.

